# Co-inoculation with *Streptomyces thermovulgaris* and commercial microbial agents enhances the reduction of antibiotic resistance genes in cattle manure composting: driving mechanisms involving microbial communities and mobile genetic elements

**DOI:** 10.3389/fmicb.2025.1688304

**Published:** 2025-11-27

**Authors:** Erguang Jin, Daoyu Gao, Yuan Zhou, Pingmin Wan, Jie Chen, Ping Gong, Peng Li

**Affiliations:** 1 Animal Husbandry and Veterinary Research Institute, Wuhan Academy of Agricultural Sciences, Wuhan, China; 2 College of Animal Science and Technology, Yangtze University, Jingzhou, China

**Keywords:** cattle manure composting, *Streptomyces thermovulgaris*, microbe community, antibiotic resistance genes, mobile genetic elements, multidrug resistant host bacteria

## Abstract

To investigate the mechanisms by which *Streptomyces thermovulgaris* a2 (Sta2) enhances the reduction of antibiotic resistance genes (ARGs) in cattle manure composting, this study compared the effects of commercial microbial inoculant (CK) and its combination with Sta2 (ST). The results showed that the ST treatment extended the thermophilic phase (≥55 °C) to 18 days (compared to 11 days with CK) and increased the removal rates of *tetG, sul1, ermQ, aac(6ʹ)-Ib-cr*, and *intI1/intI2* (by 4.8%–48.4%), simultaneously inhibiting the enrichment of *sul2* and *ermX*. During the thermophilic phase, ST treatment slowed the decline in the abundances of key genera (e.g., *Bacillus, Thermobacillus, Brachybacterium*) and effectively promoted the growth of *Actinomadura* and *Longispora* within *Actinobacteria*. Redundancy analysis revealed that bacterial community succession (56.3%) and mobile genetic elements (MGEs, 30.7%) were key drivers of ARG dynamics, with *intI1* and *Firmicutes* positively regulating most ARGs. Co-occurrence network analysis identified *Lysinibacillus* (harboring 9 ARG-MGE associations), *Luteimonas* (9), *Brachybacterium* (8), and the pathogen *Corynebacterium* (6) as multidrug resistant hosts. In summary, ST treatment enhanced the reduction of certain genes and multidrug-resistant host control by prolonging the thermophilic duration, reconstructing the microbial community composition, and effectively inhibiting *intI1*- and *intI2*-mediated horizontal gene transfer.

## 1 Introduction

The widespread use of antibiotics in animal husbandry has resulted in the continuous evolution and dissemination of drug-resistant bacteria and antibiotic resistance genes (ARGs), making livestock manure a significant reservoir of ARGs. For example, Qiu et al. (2022) detected 626 ARG subtypes across 20 classes in fecal samples, with five classes of resistance genes (*β*-lactams, multidrug, macrolides, aminoglycosides, and tetracyclines) accounting for 86.58% of the total. Once released into the environment through pathways such as rainwater runoff and manure application (Fang et al., 2018), ARGs can undergo horizontal transfer via mobile genetic elements (MGEs) like integrons and transposons or achieve vertical transmission through host proliferation (Duan et al., 2019; Zhu et al., 2023), posing threats to ecological environments and public health (Tong et al., 2022; Wang et al., 2022; Wang et al., 2023).

Aerobic composting is a common manure utilization technology. Most ARGs can be reduced through mechanisms such as high-temperature inactivation (Qian et al., 2016; Liu et al., 2023) and microbial competition (Liu et al., 2021), but the natural composting often leads to ARG residual or even enriched due to insufficient activity of indigenous microorganisms and unstable high-temperature phases (Zhu et al., 2023; Yang et al., 2024). For instance, *tetG* and *sul2* abundance increases by 20–395 times after chicken manure composting (Wang et al., 2024); tetracycline resistance genes remain highly abundant in pig manure compost products (Zhu et al., 2023).

Globally, significant efforts have been directed toward improving ARG mitigation, with notable progress in process optimization (e.g., thermal pretreatment (Zhu et al., 2023) and semi-permeable membrane coverage (Sun et al., 2024)), the addition of amendments [e.g., biochar (Tong et al., 2022) and diatomite (Wei et al., 2022)], and microbial inoculation [e.g., composite microbial agents (Li et al., 2020)]. Among these, microbial inoculation is considered a leading strategy due to its environmental friendliness and cost-effectiveness.

The addition of microbial inoculants can improve ARG removal by reconstructing the microbial community and prolonging the thermophilic phase (Li et al., 2020; Cao et al., 2020). For instance, *Bacillus megaterium* can reduce the abundance of ARGs and *sul1*/*sul2* enrichment by suppressing the activity of transposon *Tn916/1545* (Guo et al., 2020); composite microbial inoculants enhance the removal of *β*-lactam resistance genes, *tn916*, and some tetracycline resistance genes by 3.7%–23.8% (Li et al., 2020). However, research has primarily focused on *Bacillus-*dominated inoculants (Duan et al., 2019; Li et al., 2020), while studies on thermotolerant actinomycetes (e.g., *Streptomyces thermophilus*) in reducing ARGs remain scarce.

*Streptomyces thermophilus* exhibits unique advantages due to its temperature adaptability and lignocellulose degradation capabilities. Inoculation with *thermophilic Streptomyces* can enhance composting efficiency. For example, *S. thermoviolaceus* LC-10 extends the thermophilic phase and accelerates lignocellulose degradation (Zhang et al., 2024), while *Streptomyces thermovulgaris* N3C07 shortens the composting cycle (Kocak et al., 2023). *Bacillus*-*Streptomyces* synergy enhances total nitrogen content (Zhou et al., 2024), though their collaborative regulation mechanism on ARGs remains unclear. The thermophilic *Streptomyces* strains autonomously screened during the high-temperature phase of composting (>50 °C) may exhibit stronger environmental adaptability and ARG reduction potential. However, their efficacy in eliminating ARGs, as well as their cooperative mechanisms with commercial agents, still requires in-depth investigation.

Therefore, the present study applied the thermophilic *S. thermovulgaris* strain a2 (Sta2), previously isolated from the thermophilic phase (>50 °C) of cattle manure composting and identified via 16S rRNA, into a composting system. The aim was to systematically investigate the reduction mechanisms of ARGs in cattle manure composting. By comparing the treatment effects of the commercial microbial agent alone and its combination with Sta2, the study aimed to explore (1) the dynamics of bacterial community succession, ARGs and MGEs during composting; and (2) the interactions among key physicochemical parameters, bacterial communities, and MGEs in driving ARGs, along with identifying dominant driving factors. This study is the first to apply isolated Sta2 in combination with commercial microbial agents for composting, focusing on elucidating its mechanistic role in the removal of ARGs. The findings will provide practical references for optimizing microbial agents to control ARG pollution in livestock and poultry manure.

## 2 Materials and methods

### 2.1 Experimental materials

The composting materials used in this study consisted of fresh cow manure, sawdust, and broiler farm bedding (grain hulls, chicken manure, etc.). The fresh manure and sawdust were obtained from a dairy farm (Wuhan, China); the air-dried broiler farm bedding was obtained from broiler farmers (Wuhan, China). The basic characteristics of the composting materials are presented in Supplementary Table S1. Commercial microbial agent (powder form, containing *Bacillus*, *Coprococcus*, *Lactobacillus*, and *Saccharomyces* among others, with a total viable count ≥1.0 × 10^10^ CFU/g) was provided by Wuhan Xurun Environmental Protection Technology Co. Strain Sta2 was cultivated under suitable conditions, and a bacterial solution with a total bacterial load of approximately 10^9^ CFU/mL was prepared.

### 2.2 Experimental design and sample collection

The compost experiment was conducted from July to August 2022 in the rain shed of a dairy farm (Wuhan, China). An uncovered plastic box with an effective volume of 770 L was used as the compost container. The box was insulated with cotton and had evenly spaced ventilation holes (1 cm in diameter) on each side. The total weight of the raw materials used for composting was 400 kg (wet weight). A carbon to nitrogen ratio of 25:1 and a moisture content of approximately 58.0% were achieved by mixing fresh cow manure, broiler bedding, and sawdust in a ratio of 12:3:5 (wet weight). The well-mixed material was divided into two equal parts and loaded into two separate plastic boxes. Then the microbial agent was added, and the material was mixed thoroughly.

The experiment involved two groups, with one group assigned to each box. A commercial microbial agent (150 g) and pure water (600 mL) were added in the control group (labeled CK), whereas the commercial microbial agent (150 g) and Sta2 solution (600 mL) was added in the experimental treatment group (labeled ST). The compost was turned over manually on days 7, 14, 20, 25, and 30 to ensure adequate aeration. To achieve efficient composting, pure water was added on day 7 to adjust the water content to approximately 60% based on the results of water content measurements.

Representative samples were collected before pile turning on days 0 (initial feedstock, no bacterial agent), 3 (early thermophilic), 10 (mid-thermophilic), 20 (late thermophilic), and 35 (mature). A stratified, five-point sampling protocol was used: subsamples were taken from the surface of the pile at approximate depths of 10 cm (top), 30 cm (middle), and 50 cm (lower). For each layer, subsamples were combined, mixed, and homogenized via quartering. The three layer-specific samples were then pooled and underwent a final round of mixing and quartering to form a composite sample. This protocol was performed three times to obtain three technical replicates. Each sample weighed approximately 500 g and was divided into two equal portions, sealed in sterile bags, and stored at 4 °C (for physicochemical analysis) and −80 °C (for DNA analysis), respectively.

### 2.3 Measurement indicators and methods

The following determinations were repeated three times for all samples.

#### 2.3.1 Temperature measurement

Temperature was measured using a wireless temperature monitor. A metal probe was inserted 30 cm into the center of the compost material. The ambient temperature was monitored in an area without direct sunlight. Temperatures were recorded at a fixed time each day.

#### 2.3.2 pH and moisture content measurement

The pH and moisture content were determined in accordance with the methodology described by Li et al. (2020).

#### 2.3.3 Nitrate-nitrogen (NO_3_-N) content analysis

The samples were extracted using 2 mol/L potassium chloride. The resulting extract was diluted according to a standard curve, and the NO_3_-N content was analyzed using a flow analyzer (AA3, SEAL Analytical, UK).

### 2.4 DNA extraction and quantitative polymerase chain reaction (qPCR)

Total DNA was extracted from samples using the FastDNA^®^ Spin Kit for Soil (MP Biomedical, USA). The purity and concentration of DNA were determined using a Nanodrop ONE^C^ spectrophotometer (Thermo Fisher Scientific, USA), and all extracts were of high quality (A260/A280 = 1.82 ± 0.02). The DNA was stored at −80 °C, and the relevant concentration data are presented in Supplementary Table S2.

The analysis targeted 22 genes, covering 7 tetracycline genes (*tetB/P*, *tetC*, *tetG*, *tetQ*, *tetT*, *tetW*, and *tetX*), 3 sulfonamide genes (*sul1*, *sul2*, and *dfrA7*), 2 macrolide genes (*ermQ* and *ermX*), 5 quinolone genes (*gyrA*, *parC*, *qnrA*, *qnrC*, and *qnrS*), 1 aminoglycoside gene (*aac(6′)-Ib-cr*), and 4 MGEs (*intI1*, *intI2*, *Tn916/1545*, and *ISCRI*) (Supplementary Table S4). This selection was based on their clinical relevance in dairy farming, documented environmental prevalence, and the role of MGEs in horizontal gene transfer (Oliver et al., 2020; Zhang et al., 2020; Qiu et al., 2022).

The target genes were screened by conventional PCR using gene-specific primers (Tsingke Biotechnology Co., Ltd., Beijing, China; sequences in Supplementary Table S3). Amplification products were verified by 1.5% agarose gel electrophoresis, and only samples with a single band of the correct size were analyzed by qPCR (Supplementary Table S4). Quantification of the target and 16S rRNA genes was performed in triplicate using a Bio-Rad CFX Connect™ real-time PCR system with DNA templates uniformly diluted to 10 ng/μL. Reaction specificity was verified by melting curve analysis. The standard curve showed a correlation coefficient (*R*^2^) ≥ 0.99, with the detection limit set at Ct = 31. The relative abundance of the target gene was calculated via the 2^(–ΔΔCt) method, using the 16S rRNA gene as the internal reference. Detailed reaction systems and thermal cycling conditions are provided in the Supplemental material (Liu et al., 2023; Li et al., 2020).

### 2.5 Amplicon sequencing

The V3-V4 region of the bacterial 16S rRNA gene was amplified using primers 341F/805R and sequenced on an Illumina MiSeq platform by Wuhan Benetech Co., Ltd. Raw sequencing reads were quality-filtered using Trimmomatic (v0.39). Subsequent processing within QIIME2 (v2022.3) involved denoising, paired-read merging, and chimera removal with the DADA2 plugin to generate amplicon sequence variants (ASVs). The ASV table was then filtered to remove rare ASVs and rarefied to the minimum sequencing depth for downstream statistical analysis.

### 2.6 Data processing and analysis

Data were analyzed using Microsoft Excel (2016), SPSS 25.0, and R (v4.2.2). Analyses included: t-tests for within-group differences of target genes; DESeq2 for differential microbial abundance; correlation analyses (Spearman/Pearson), and redundancy analysis (CANOCO 5.1). Principal coordinates analysis based on Bray–Curtis distance was conducted to examine community structure variation. Visualizations were created with GraphPad Prism 8.0.2 (trend lines), RAW Graphs 2.0 and Paysono platforms (heatmaps), and Gephi 0.9.2 (network diagrams).

## 3 Results

### 3.1 Changes in physicochemical parameters

**Figure 1:**
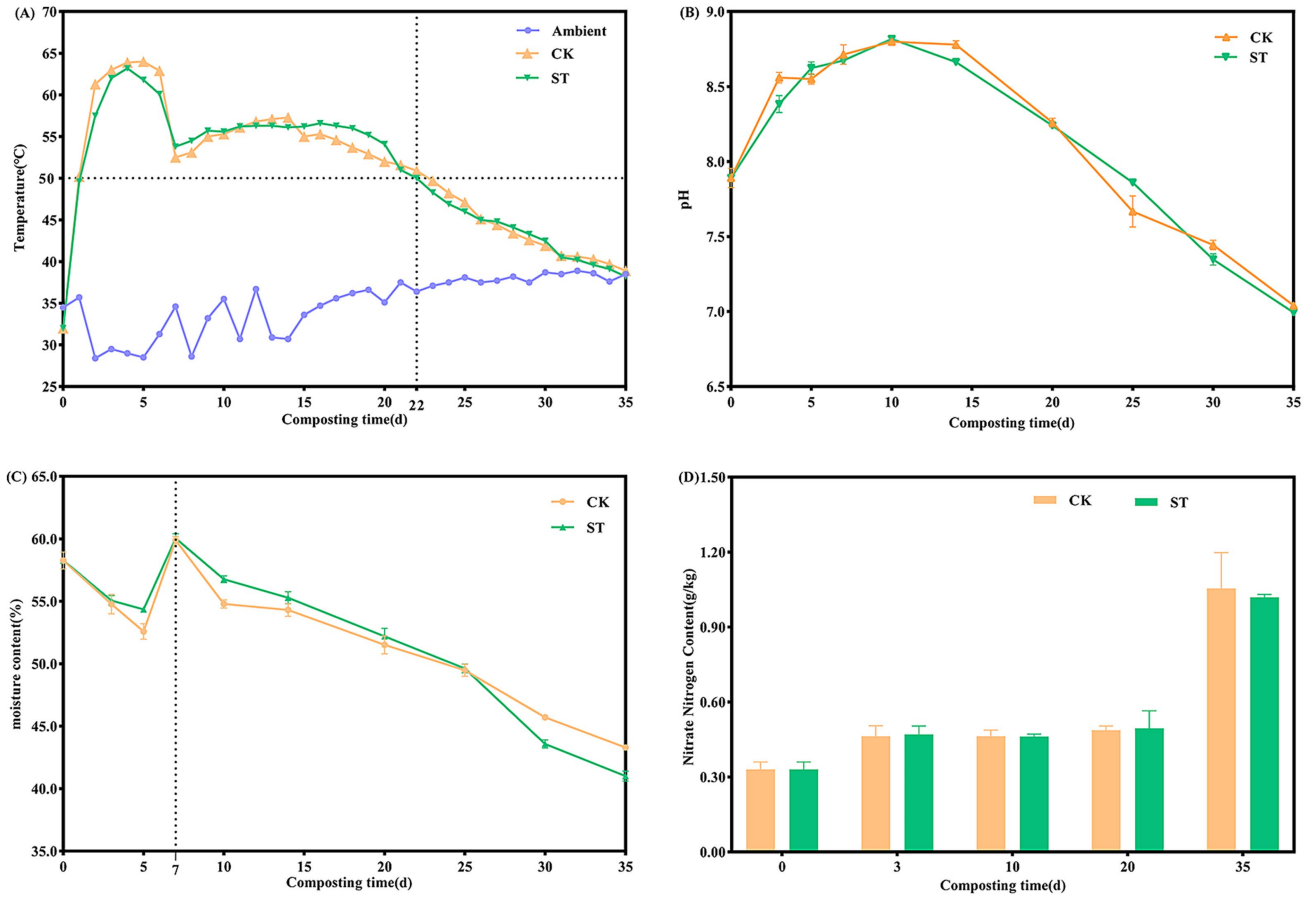
Changes in physicochemical parameters during composting: **(A)** temperature (on-line monitoring), **(B)** pH, **(C)** moisture content, and **(D)** NO₃-N content. Data points in **B–D** represent the mean of triplicate replicates, and error bars indicate the standard deviation.

The trends in characteristic physicochemical parameters during the composting process are illustrated in Figure 1. The temperatures of the two treatments rapidly increased to approximate 50 °C on day 1, remained above 50 °C on days 2–22, and then gradually decreased to ambient temperature (Figure 1A). The ST group sustained temperatures above 55 °C for 18 days, exceeding this duration for the CK group by 7 days and thereby meeting the sanitary requirements for fecal treatment. Both treatments exhibited a unimodal pH pattern (Figure 1B), peaking at approximately 8.8 on day 10 and declining gradually to ~7.0 by the end of compositing. Moisture content displayed an overall decreasing trend (Figure 1C), with a rapid decline from 0 to 5 days and a transient increase after moisture adjustment on day 7. The final moisture content decreased from 58.3% to 43.3% (CK) and 41.0% (ST), with the ST group achieving a 15.3% higher evaporation efficiency. The NO₃-N content (Figure 1D) exhibited stage-specific accumulation, initiated at 0.3 g/kg. It increased gradually during the first 20 days, followed by rapid accumulation from day 20–35, ultimately reaching 1.1 g/kg (CK) and 1.0 g/kg (ST).

### 3.2 Changes in ARGs and MGEs

**Figure 2:**
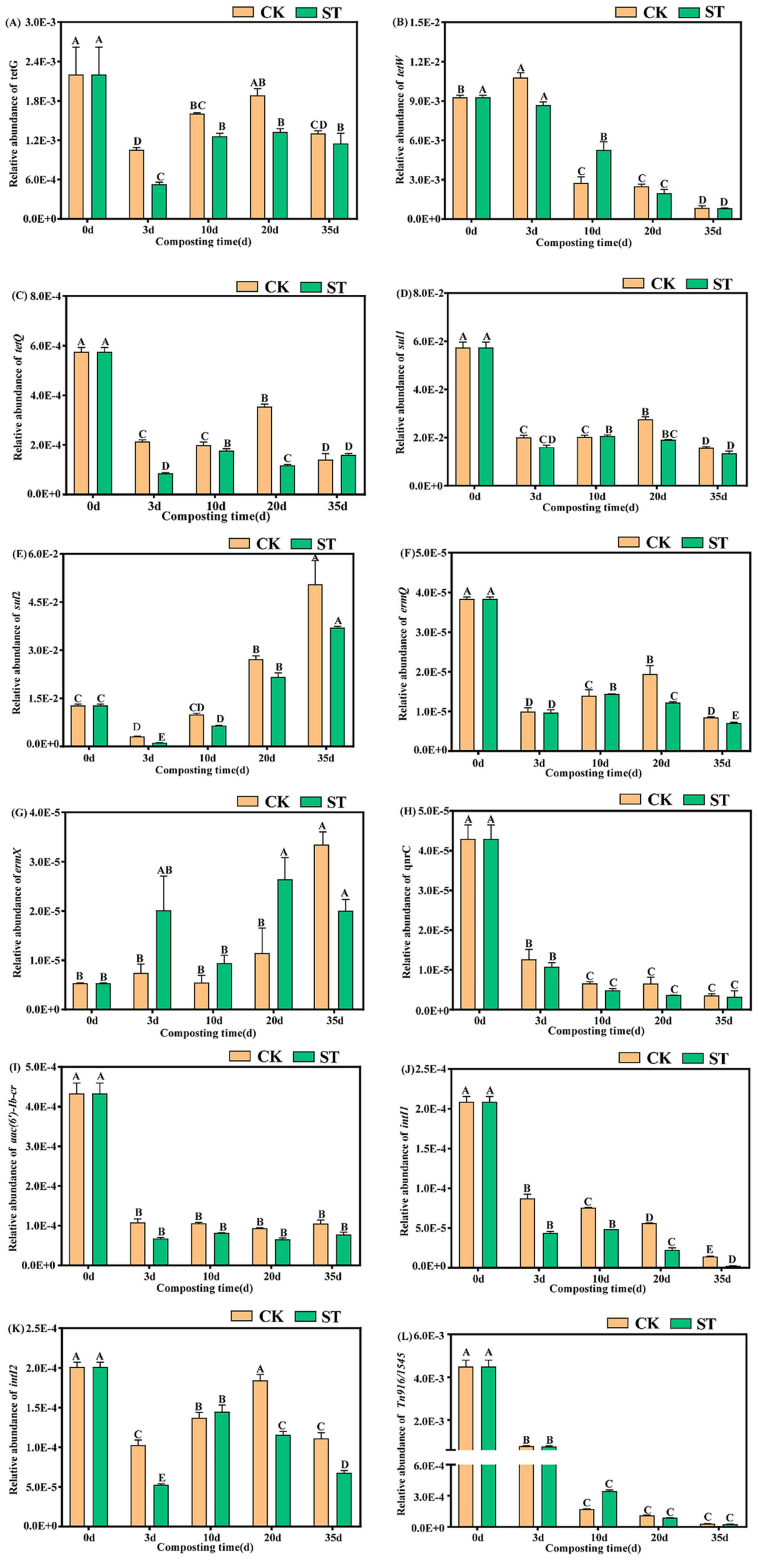
Changes in the relative abundances of ARGs and MGEs during composting. Different capital letters within the same group indicate significant differences (*p* < 0.01).

A total of 12 target genes were detected in the composting feedstock, including tetracycline (*tetG*, *tetW*, and *tetQ*), sulfonamide (*sul1* and *sul2*), macrolide (*ermQ* and *ermX*), quinolone (*qnrC*), aminoglycoside (*aac(6′)-Ib-cr*), as well as MGEs (*intI1*, *intI2*, *Tn916/1545*). These showed varying change trends during composting (Figure 2). Tetracycline ARGs (*tetG*, *tetQ*, *tetW*) generally decreased, with *tetW* showing a near-continuous decline (Figures 2A–C). Among the sulfonamide ARGs, *sul1* decreased overall, whereas *sul2* dropped during the high-temperature phase (days 3–10) before rebounding to exceed its initial level (Figures 2D,E). For other ARGs, *ermQ* exhibited a “decrease–increase–decrease” fluctuation pattern; *qnrC* and *aac(6′)-Ib-cr* underwent continuous attenuation; and *ermX* increased persistently (Figures 2F–I). Of the MGEs, *intI1* and *Tn916/1545* exhibited sustained overall declines, while *intI2* decreased with fluctuations (Figures 2J–L).

At the end of composting, the RAs of *tetG*, *tetW*, *tetQ*, *sul1*, *ermQ*, *qnrC*, and *aac(6′)-Ib-cr* decreased significantly by 40.7%–91.6% in the CK and 47.7%–92.5% in the ST compared to initial levels (*p* < 0.01). Similarly, *intI1*, *intI2*, and *Tn916/1545* decreased by 44.6%–98.8% (CK) and 66.3%–99.4% (ST) (*p* < 0.01). In contrast, *sul2* increased by 3.9-fold (CK) and 2.9-fold (ST) (*p* < 0.01), while *ermX* increased by 6.3-fold (CK) and 5.0-fold (ST) (*p* < 0.01). Compared with CK, the ST group exhibited 4.8%–17.2% greater removal of *tetG*, *sul1*, *ermQ*, and *aac(6′)-Ib-cr*, and 6.6% and 48.4% greater removal of *intI1* and *intI2*, respectively. Concurrently, the enrichment of *sul2* and *ermX* in ST were lower than those in CK (by 1.0-fold and 1.3-fold, respectively). In conclusion, the ST group enhanced the reduction of ARGs and MGEs, while effectively suppressing the enrichment of *sul2* and *ermX*.

### 3.3 Changes in bacterial communities

**Figure 3:**
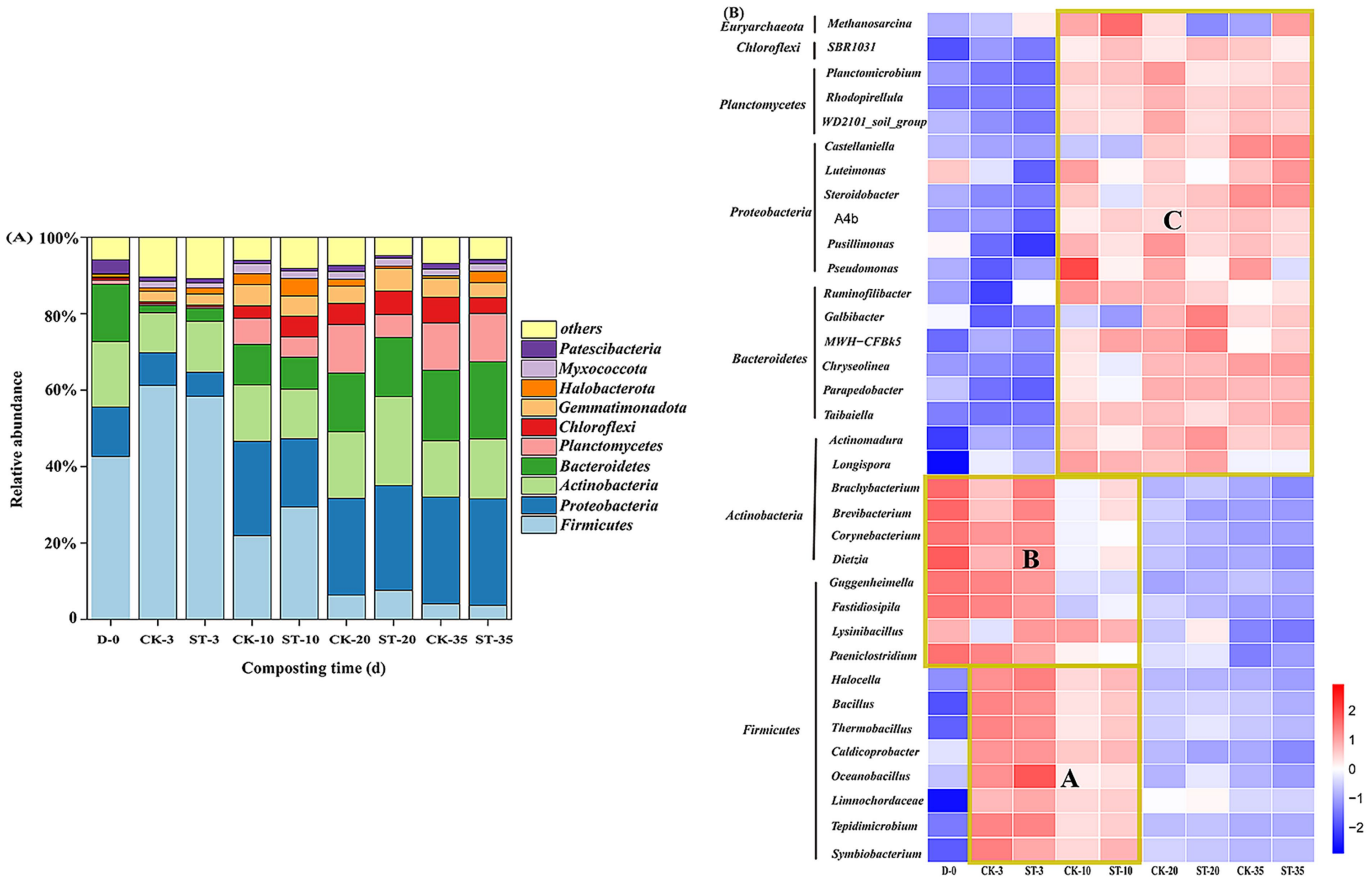
Changes in the relative abundances of the dominant phyla **(A)** and genera **(B)** during compositing (based on the mean values of three technical replicates).

Principal coordinates analysis (PCoA) based on Bray–Curtis distance revealed that PC1 and PC2 together explained 87.5% of the variation (Supplementary Figure S1). Samples from the same time points formed distinct clusters according to sampling time: 0-day samples formed a distinct cluster; 3-day and 10-day samples exhibited higher dispersion; and 20-day and 35-day samples showed the closest proximity. At the phylum level (top 10), the community exhibited a temperature-driven, staged succession (Figure 3A). At the initial stage (0 days), the community was dominated by *Firmicutes* (42.6%), *Actinobacteria* (17.2%), *Bacteroidetes* (15.0%), and *Proteobacteria* (12.9%), collectively accounting for 87.7% (Supplementary Table S5). In the early high-temperature phase (3 days), the RA of *Firmicutes* increased (CK 61.2%, ST 58.4%) (*p* < 0.05 vs. day 0), while those of the other three major phyla decreased. Subsequently, the *Firmicutes* abundance decreased progressively, while the *Proteobacteria*, *Actinobacteria*, and *Bacteroidetes* increased. Concurrently, *Planctomycetes* also exhibited substantial proliferation. By the late high-temperature phase (20 days), the microbial community structure had shifted to be dominated by these four phyla (70.8%–72.2%). Compared with the CK group, the ST group exhibited a smaller decrease in *Firmicutes* abundance (−50.8% vs. -54.8%) and a greater increase in *Actinobacteria* (+9.9% vs. +6.9%) during the thermophilic phase, while the increase in *Planctomycetes* was 6.7 percentage points lower (*p* > 0.05 for all comparisons). During the late composting stage (20–35 days), the ST group displayed a 5.0-percentage-point greater reduction in *Actinobacteria* than the CK group (*p* > 0.05). At the conclusion of composting, relative to initial levels, the abundance of *Firmicutes* (CK: −38.5%, ST: −38.9%; *p* < 0.05) and *Actinobacteria* (CK: −2.4%, ST: −1.5%; *p* < 0.05) declined, while those of *Proteobacteria* (CK: +15.0%, ST: +14.9%; *p* > 0.05), *Bacteroidetes* (CK: +3.5%, ST: +5.2%; *p* < 0.05), and *Planctomycetes* (CK: +11.3%, ST: +11.6%; *p* < 0.05) rose. The abundance of *Chloroflexi* increased to 6.7% (CK) and 4.1% (ST) (*p* < 0.05) (Supplementary Table S5).

At the genus level (top 35), the microbial communities clustered into three groups (A, B, and C) based on their abundance profiles across different composting phases (Figure 3B). Cluster A, comprising eight *Firmicutes* genera (e.g., *Bacillus*, *Thermobacillus*), increased sharply from 1.8% to 73.1%–78.5% (*p* < 0.001) by day 3, then declined to 5.4–6.9% (*p* < 0.001) by the end of composting (lower in the ST group, *p* > 0.05) (Supplementary Table S6). On day 10, the ST group abundance (39.1%) was higher than that of the CK group (28.9%) (*p* > 0.05). By day 20, four genera (e.g., *Bacillus*, *Thermobacillus*) within this cluster maintained higher proportions in the ST group than in the CK group. Cluster B, including four *Firmicutes* (e.g., *Lysinibacillus*) and four *Actinobacteria* genera (e.g., *Corynebacterium*), initially dominated (79.6%; 62.2% contributed by *Actinobacteria*) but declined to 1.1%–1.5% (*p* < 0.001) by the end (Supplementary Table S6), with Corynebacterium decreasing by >98.7% (*p* < 0.05). The ST group exhibited higher abundances of specific genera: on day 10, *Actinobacteria* genera and *Fastidiosipila* were 1.7- and 2.3-fold more abundant than in CK, respectively; by day 20, *Lysinibacillus* (1.4% vs. 0.6%, *p* < 0.05) and *Brachybacterium* (0.3% vs. 0.2%, *p* > 0.05) remained more abundant. Cluster C, comprising 19 genera from *Bacteroidetes*, *Proteobacteria*, *Chloroflexi,* and *Euryarchaeota*, increased from 18.5% to 91.6–93.5% (*p* < 0.001) by the end (Supplementary Table S6). Notably, key initial genera within this cluster, including *Luteimonas* (4.7%), *Pusillimonas* (2.6%), *Pseudomonas* (2.7%), and *Methanosarcina* (3.6%), exhibited divergent successional patterns: *Luteimonas* and *Pusillimonas* exhibited no substantial increase, whereas *Pseudomonas* abundance increased by 2.7-fold (CK, *p* < 0.05) and 0.41-fold (ST, *p* > 0.05) relative to initial levels. *Methanosarcina* peaked on day 10 (CK: 7.7%; ST: 11.4%, *p* > 0.05) then declining to 2.1% (CK) and 9.8% (ST) (*p* < 0.05). *Actinomadura* and *Longispora* increased to 12.6% (CK) and 19.5% (ST) (*p* < 0.001) on day 20 (Supplementary Table S7), and ultimately reached 26.1-fold (CK) and 54.5-fold (ST) (*p* < 0.001) of the initial abundance. Initially rare genera (abundance <0.2%), such as *Steroidobacter* (10.8%–11.4%), *Castellaniella* (7.4%–7.8%), and *Chryseolinea* (23.6%–25.1%) (*p* < 0.05), emerged as dominant taxa by the end (Supplementary Table S7). Additionally, the inoculated strain Sta2 was not detected in any samples. Overall, temperature dynamics drove significant microbial succession.

### 3.4 Correlation among microbiota, MGEs, physicochemical parameters, and ARGs

**Figure 4:**
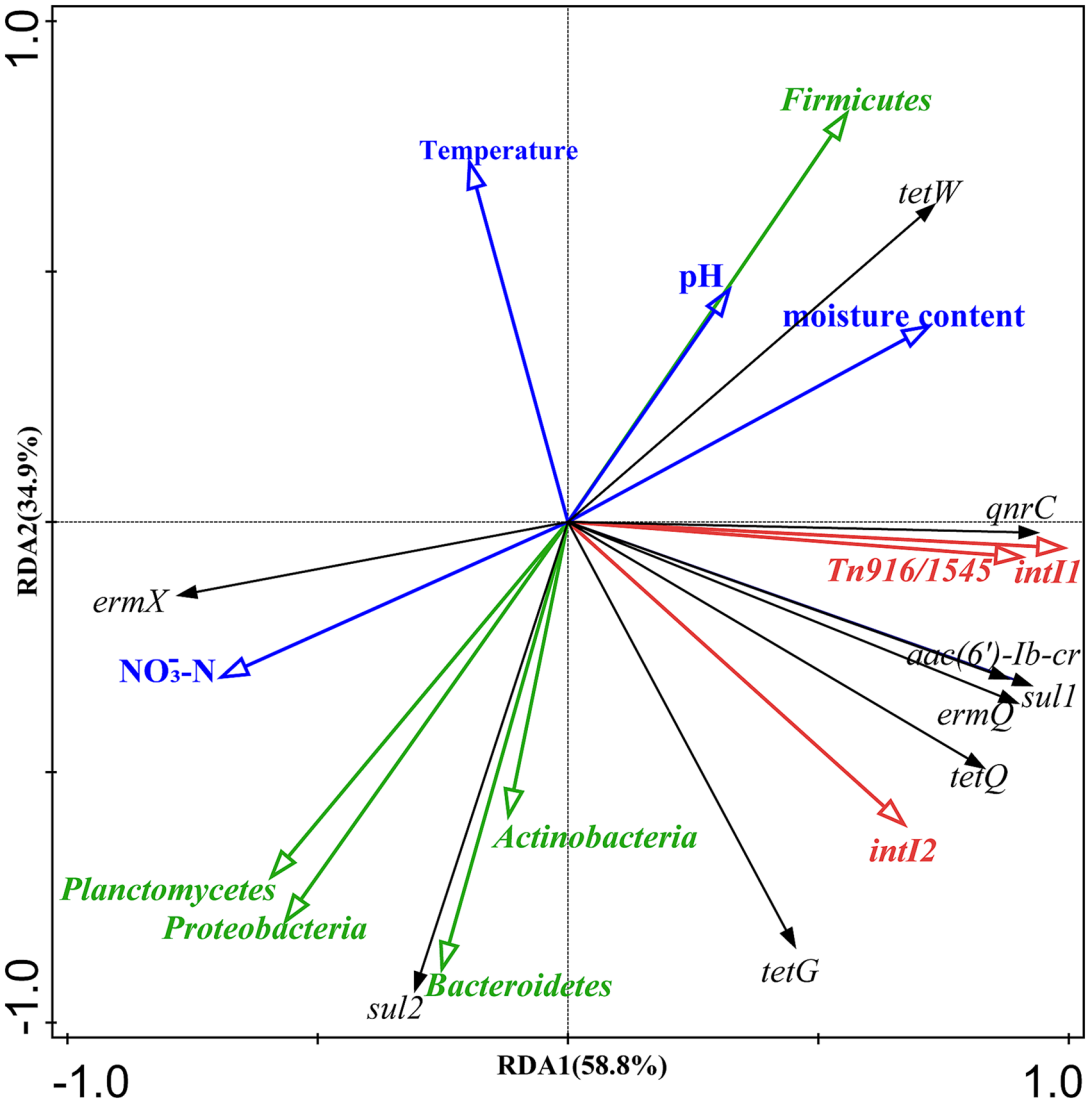
Redundancy analysis of bacterial phyla, physicochemical properties, ARGs and MGEs. The arrows represent different variable groups: bacterial phyla (green), physicochemical parameters (sky blue), ARGs (black), MGEs (red).

Redundancy analysis (RDA) was performed to analyze the effects of physicochemical parameters (pH, temperature, moisture, and NO_3_-N), the bacterial community (at the phylum level), and MGEs (*intI1*, *intI2*, and *Tn916/1545*) on the dynamics of ARGs during composting (Figure 4). The results showed that these variables collectively explained 93.7% of the total variation in ARGs (RDA1 58.8%, RDA2 34.9%). The bacterial community exhibited the highest contribution (56.3%), followed by MGEs (30.7%), while physicochemical parameters had the lowest contribution (6.7%). The bacterial community and MGEs collectively explained 87.0% of the variation in ARGs, with *intI1* and *Firmicutes* responsible for 49.3% and 25.6% of the variation, respectively.

Spearman correlation analysis revealed distinct association patterns between bacterial phyla and ARGs/MGEs (Supplementary Figure S2). *Firmicutes* showed positive correlation with most targets, exhibiting significant positive correlations with *tetW*, *qnrC*, and MGEs (*intI1*, *Tn916/1545*) (*p <* 0.01)*. Actinobacteria* was significantly positively correlated with *tetG* and *sul2* (*p <* 0.01), and with *intI2* (*p* < 0.05). Although generally negatively correlated with most genes, *Proteobacteria* showed significant positive correlations with *sul2* and *ermX* (*p* < 0.01)*. Bacteroidetes* was significantly positively correlated solely with *sul2* (*p* < 0.01).

Pearson correlation analysis (Supplementary Figure S3) revealed specific associations between MGEs and ARGs. *intI1* and *Tn916/1545* showed significant positive correlations (*p* < 0.01) with five ARGs (e.g., *tetQ, sul1*); *intI2* was significantly positively correlated (*p* < 0.01) with *tetG*, *tetQ,* and *ermQ*. MGEs were overall negatively correlated with *sul2* and *ermX*, with a significant negative correlation between *intI1* and *ermX* (*p* < 0.05). Moreover, *sul2* and *ermX* showed weak, non-significant negative correlations (*p* > 0.05) with most other ARGs.

Regarding physicochemical parameters (Supplementary Figure S2), temperature was negatively correlated with most genes, showing highly significant correlations with *tetG*, *sul2*, *aac(6′)-Ib-cr*, and *intI2* (*p* < 0.01). pH correlated negatively with sul2 (*p* < 0.01) and positively with *tetW* (*p* < 0.05). Moisture content showed significant positive correlations, whereas NO₃-N showed significant negative correlations, with the same five ARGs (e.g., *tetW*, *sul1*) and the three MGEs (*p* < 0.01).

### 3.5 Co-occurrence network of ARGs, MGEs, and microbial communities

**Figure 5:**
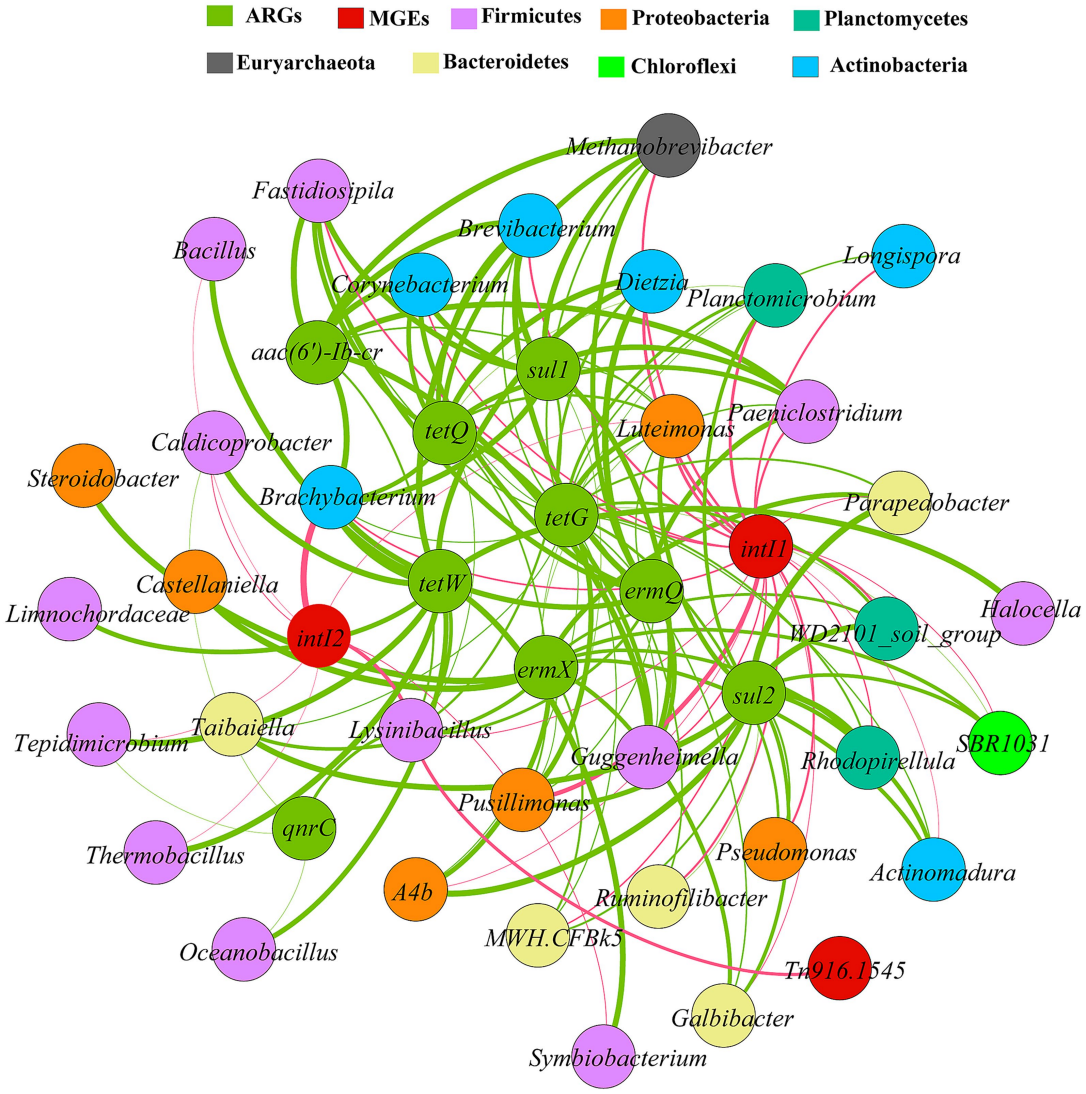
Network analysis of the co-occurrence of ARGs, MGEs and potential host bacteria. Connecting lines indicate significant positive correlations (*p* < 0.05) according to Spearman correlation coefficients (*R* > 0.8). The thickness of the line represents the magnitude of the correlation.

Spearman correlation analysis (R > 0.8, *p* < 0.01) was used to construct a co-occurrence network among nine ARGs, three MGEs, and the top 35 most abundant bacterial genera (Figure 5). The results revealed significant positive correlations (*p* < 0.01) between all bacterial genera and the target genes. Based on co-occurrence patterns, *Firmicutes* (12 genera), *Proteobacteria* (6 genera), *Actinobacteria* (6 genera), and *Bacteroidetes* (5 genera) could represent the primary potential host phyla, collectively accounting for 82.9% of the associated genera. The number of genera associated with each gene varied widely: *intI1* and *tetG* (24 each), *sul2* and *ermX* (15 each), *tetW* (13), *qnrC* (4).

Multi-gene co-occurrence was prevalent. For example, *Lysinibacillus* was associated with nine genes: *tetW*, *tetQ*, *sul1*, *qnrC*, *ermQ, aac(6′)-Ib-cr, intI1, intI2,* and *Tn916/1545*. Similarly, *Luteimonas* was also associated with nine genes: *tetG, tetQ, sul1, sul2, ermX, ermQ, aac(6′)-Ib-cr, intI1,* and *intI2*, while *Brachybacterium* and *Brevibacterium* were linked to eight and seven genes, respectively. Furthermore, *ermX* and *sul2* exhibited highly overlapping associated genera (sharing 13 out of 15), distributed across *Bacteroidetes*, *Planctomycetes*, and *Proteobacteria*. The integrons *intI1* and *intI2* were associated with 24 and 8 genera, respectively, and the transposon *Tn916/1545* with one genus. Notably, *Lysinibacillus* was linked to three MGEs and *Luteimonas* to both integrons. Associations involving human pathogens were also identified: *Corynebacterium* with *tetG, tetQ, sul1, ermQ, aac(6′)-Ib-cr,* and *intI1*; *Pseudomonas* with *tetG, sul2,* and *intI2*; and *Bacillus* with *tetW* and *intI2*.

## 4 Discussion

In this study, co-inoculation extended the thermophilic phase (>55 °C) to 18 days, outperforming the 12-day duration achieved by single *S. thermophilus* inoculation (Zhang et al., 2024). This enhanced performance can be attributed to the synergistic interaction between the commercial inoculant and strain Sta2. Specifically, the unique role of Sta2 lies not in self-proliferation but in its synergistic stimulation with the commercial inoculant, which prompted targeted thermophile enrichment (Zhang et al., 2024). The resultant sustained microbial activity facilitated continuous organic matter decomposition and heat generation (Wei et al., 2019; Dong et al., 2024), thereby enhancing moisture evaporation in the ST group. During the cooling/maturation phase, the rapid accumulation of NO_3_-N aligned with the restored activity of nitrifying bacteria following temperature decrease (Yuan et al., 2018). The initial increase in pH, driven by ammonia released from organic matter decomposition, was followed by a gradual decline due to nitrification and organic acid accumulation (Liu et al., 2021). Collectively, these results indicate that the ST treatment enhanced the composting efficiency by extending the thermophilic phase, intensifying organic matter decomposition, and optimizing nitrogen transformation.

The findings demonstrate that addition of microbial agents during composting effectively remove most of the ARGs and MGEs (Li et al., 2020; Cao et al., 2020). Specifically, the reductions in tetracycline resistance genes align with literature (Duan et al., 2019). The lower removal of *tetG* (40.7%–47.7%) may be attributed to its efflux pump mechanism (Guo et al., 2019) and to the persistence of potential host bacteria (e.g., *Actinomadura* and *Pseudomonas*), which significantly correlated with *tetG* abundance (Figure 5). Sulfonamide resistance genes (*sul1/sul2*) declined primarily during the thermophilic phase, underscoring its importance for their removal (Qian et al., 2016). The persistent attenuation of *sul1* may stem from thermal inactivation of host bacteria (Sun et al., 2024), while later-stage *sul2* enrichment could be related to its broad host adaptability (Wei et al., 2022) or the presence of thermophilic hosts (Sun et al., 2024). The persistent declines in *aac(6′)-Ib-cr, ermQ*, and *qnrC* are also consistent with previous research (Wei et al., 2022; Li et al., 2022). In contrast, *ermX* enrichment may primarily stem from vertical transmission during host bacterial proliferation (Wang et al., 2021).

The significant reductions in three MGEs align with previous research findings (Liu et al., 2021; Cao et al., 2020). However, the removal efficiency of *intI2* remained moderate (44.6%–66.3%), which may be associated with specific microbial community succession (Tong et al., 2022). This persistent *intI2* may promote horizontal gene transfer, increasing the risk of ARG dissemination and potentially leading to the rebound of certain ARGs (Liu et al., 2023). Furthermore, while the set of target genes used was representative, it was not exhaustive. Future studies could employ expanded screening or metagenomics to achieve broader coverage of ARGs, including *β*-lactamases.

Collectively, microbial inoculants likely enhance ARG reduction by promoting the succession of the microbial community, accelerating organic matter degradation (Li et al., 2020; Cao et al., 2020), and thermally inactivating hosts or degrading ARGs (Chen et al., 2019). Notably, the ST group exhibited an enhanced effect, achieving greater removal efficiencies for several targets (particularly *intI2*) while also curbing the enrichment of *sul2* and *ermX*. These results demonstrate that combining Sta2 with commercial microbial agents enhanced the reduction of resistance genes, as also reported for a composite microbial inoculant by Li et al. (2020). The primary mechanism may involve the introduction of Sta2 stimulating the proliferation of thermotolerant bacteria, which alters the community structure and prolongs the thermophilic phase via sustained organic matter degradation, leading to efficient killing or competitive inhibition of host bacteria (Wei et al., 2019; Duan et al., 2019; Wang et al., 2021). Concurrently, Sta2 inhibits the activity of *intI1/intI2* to impede horizontal gene transfer, as previously observed (Duan et al., 2019; Liu et al., 2021). Thus, combining Sta2 with commercial inoculants is a viable strategy for enhancing the reduction of ARGs/MGEs while mitigating the enrichment of persistent ARGs.

The succession of microbial communities during composting displays temperature-driven phasic characteristics, supporting temperature as a key driver of microbial succession (Zhang et al., 2020; López et al., 2021). The greater dispersion during the thermophilic phase likely reflects the differential effects of the inoculants, whereas the convergence of samples during the cooling/maturation phase indicates the establishment of a stable microbial community structure.

At the phylum level, the initial dominance of *Firmicutes*, *Actinobacteria*, *Bacteroidotes,* and *Proteobacteria* agrees with typical cattle manure composition (Zhang et al., 2020). The thermophilic nature of *Firmicutes* accounts for its rapid proliferation during the early high-temperature phase (López et al., 2021), which may have competitively inhibited other phyla (Tong et al., 2022). Subsequent succession, characterized by a decreasing *Firmicutes* and increasing in *Actinobacteria, Bacteroidetes*, and *Proteobacteria*, followed the established trend reported in previous studies (Hu et al., 2019; Li et al., 2020). The late-stage increase of *Chloroflexi* is associated with its thermophilic nature (Wang et al., 2021). The functional importance of these phyla is well-established: *Actinobacteria*, *Bacteroidetes*, *Proteobacteria*, and *Chloroflexi* are recognized as key participants in lignocellulose degradation (Guo et al., 2021; Li et al., 2023), whereas *Proteobacteria* and *Planctomycetes* play crucial roles in nitrogen transformation (Tong et al., 2022; Zhou and Yao, 2020). Their collective rise in later stages suggests synergistic promotion of these processes. The introduction of Sta2 influenced community dynamics: during the thermophilic phase, it was associated with a smaller reduction in *Firmicutes* and a greater increase in *Actinobacteria* compared to CK group, indicating that Sta2 may promote actinobacterial proliferation (Liu et al., 2022). The concurrent smaller increase in *Planctomycetes* may result from competitive inhibition by Sta2 metabolites (Zhang et al., 2024). Furthermore, *Actinobacteria* underwent a greater reduction in the ST group during the cooling/maturation phase, likely because the introduction of Sta2 prolonged the high-temperature period, which accelerated organic matter decomposition and microbial succession (Dong et al., 2024). The final dominance of *Proteobacteria*, *Bacteroidetes*, and *Planctomycetes* indicating compost maturation (Li et al., 2020; Zhou and Yao, 2020).

Genus-level cluster analysis clarified the successional trajectory of functional microbial communities. The rapid proliferation of Cluster A genera (e.g., *Bacillus, Thermobacillus*) in the first 3 days was likely stimulated by exogenous microbial agents, enhancing microbial activity (Guo et al., 2020), promoting decomposition of organic matter and temperature rise (Zhou et al., 2019; Cao et al., 2020), and driving *Firmicutes* abundance dynamics. The sustainedly higher abundance of thermophilic genera (e.g., *Bacillus*, *Thermobacillus*) in the ST group during the high-temperature phase may be attributed to the extended high temperatures favoring their growth (Zhou et al., 2019), while its lower final abundance was likely due to the recolonization of mesophilic microorganisms and fungi during the cooling/maturation phase (Ezugworie et al., 2021).

The initial high abundance of Class B, particularly of *Actinobacteria* genera, is crucial for the transformation of organic compounds in the early stages, especially lignocellulose decomposition (Liu et al., 2022). Its significant decrease by the final phase, particularly *Corynebacterium*, was likely due to thermophilic-phase inhibition of mesophilic genera (Liu et al., 2020). The higher abundances of specific functional genera (e.g., *Brachybacterium*, *Lysinibacillus*) in the ST group during the thermophilic phase suggest that Sta2 may support the survival of these taxa by enhancing enzymatic activity and reshaping the microbial community (Zhao et al., 2017; Wei et al., 2019).

The dominance of Class C in the mid-to-late stages drove organic matter transformation and humification. Initially high-abundance genera (e.g., *Luteimonas*, *Pseudomonas*) likely originated primarily from feedstock, while their persistence may be linked to thermotolerance (Zhang et al., 2020; Li et al., 2023). *Actinomadura* and *Longispora* emerged as the primary *Actinobacterial* contributors in mid-late stages, effectively decomposing recalcitrant compounds (Wei et al., 2019). Their consistently higher abundance in the ST group suggests that Sta2 may have promoted their proliferation (Liu et al., 2022). Conversely, the smaller increase in *Pseudomonas* in the ST group may reflect inhibition from the prolonged thermophilic phase (Liu et al., 2020).

Moreover, several initially rare genera were enriched as dominant taxa by the conclusion of composting, such as *Steroidobacter* and *Castellaniella* for nitrogen transformation (Tong et al., 2022), and *Chryseolinea* for macromolecule degradation (Guo et al., 2021). This enrichment is imperative for compost maturation. Notably, the sustained higher abundance of *Methanosarcina* in the ST group may possibly be due to a positive effect of Sta2 introduction on its growth or local anaerobic microenvironments, though the responsible mechanisms require further study. In summary, Sta2 likely accelerates maturation by synergistically sustaining thermophilic-phase activity of heat-resistant bacteria (e.g., *Bacillus*, *Thermobacillus, Brachybacterium*), enhancing cellulose degraders (e.g., *Actinomadura*, *Longispora*), and driving functional microbiota succession (e.g., *Castellaniella*) in the maturation phase (Dong et al., 2024). The absence of the inoculated strain Sta2 in the sequencing data implies a lack of successful colonization, supporting its role as an “ecological initiator” that regulates indigenous microbes rather than proliferates itself (Zhang et al., 2024). Thus, this mode of action presents an inherently low risk for ARG dissemination. The genomic characterization of strain Sta2 remains a target for future research.

RDA revealed that the dynamic changes of ARGs during composting were primarily driven by microbial community succession, MGEs, and physicochemical parameters, which is consistent with previous studies (Wang et al., 2022). Among these factors, microbial communities exhibited the highest contribution rate, indicating their role as the primary driver of ARG evolution (Zhang et al., 2020; Zhao et al., 2021). Bacterial communities and MGEs collectively explained the vast majority of the variation in ARGs, directly implicating them as drivers (Liu et al., 2021). Notably, *intI1* and the phylum *Firmicutes* exhibited the highest independent explanatory power for ARG variation, suggesting their crucial role in influencing ARG dynamics (Duan et al., 2019). The significant positive correlations of *Firmicutes* with *tetW*, *qnrC*, and key MGEs indicate that changes in this phylum may influence the dynamics of these genes and that specific taxa within it could be potential hosts (Qiu et al., 2022; Wang et al., 2024). The substantial decrease in *Firmicutes* abundance post-composting, especially in the ST group, was likely the primary factor causing the reductions in ARGs/MGEs and the enhanced removal efficiency of specific genes in the ST group (Duan et al., 2019). Similarly, *Actinobacteria*, significantly positively correlated with *tetG*, *sul2*, and *intI2* might be associated with their persistence. Conversely, the increased abundance of *Proteobacteria* and *Bacteroidetes* during the late composting stage supported the maintenance of *sul2* and *ermX*, aligning with their respective significant positive correlations.

The high contribution of MGEs to the variation of ARGs, particularly *intI1*, highlights their crucial role in ARG dynamics (Tong et al., 2022). The significant positive correlations of *intI1* and *Tn916/1545* with five ARGs, as well as of *intI2* with *tetG, tetQ,* and *ermQ,* indicate that MGEs may play pivotal roles in the horizontal transfer of these ARGs (Tong et al., 2022; Li et al., 2022). The overall negative correlation between MGEs and *sul2/ermX* implies that horizontal transfer may not be the primary driver for these two genes (Wang et al., 2024). The significant post-composting reduction in MGE abundance confirms that inhibition of MGE-mediated horizontal transfer can effectively reduce ARGs (Wei et al., 2022; Zhu et al., 2023). The greater reductions of *intI1* and *intI2* in the ST group suggest that Sta2 may suppress MGE proliferation, consistent with the reported effect of microbial agents on *intI1* (Duan et al., 2019). Furthermore, the weak, non-significant correlations of *sul2* and *ermX* with most other ARGs indicate that their enrichment did not impede the removal of other genes, possibly due to differential ARG responses and host adaptation strategies during composting (Cao et al., 2020; Wang et al., 2024).

Moreover, physicochemical parameters can indirectly influence ARG dynamics by regulating microbial activity (Sun et al., 2024). The negative associations of temperature with most ARGs/MGEs suggest it may act as a primary driver of ARG variation (Ma et al., 2023; Sun et al., 2024). pH exhibited gene-specific effects, with its positive correlation with *tetW* and negative correlation with *sul2*, consistent with prior studies (Duan et al., 2019; Zhang et al., 2024). Moisture content positively correlated with multiple ARGs, aligning with the results of Zhang et al. (2020). Meanwhile, NO₃-N showed negative associations, which may be related to its indirect effects on potential host bacteria (Wang et al., 2020). These findings demonstrate that physicochemical parameters collectively shape ARG fate during composting by driving microbial succession (Liu et al., 2021). In summary, ARG reduction during composting is a synergistic process dominated by microbial community succession (Li et al., 2022), mediated by horizontal gene transfer via MGEs (Tong et al., 2022), and modulated by key physicochemical parameters (Wang et al., 2020). The introduction of Sta2 represents a promising strategy for reducing relevant ARGs through a greater reduction in *Firmicutes* abundance and suppressing MGE activity (Duan et al., 2019; Tong et al., 2022).

Co-occurrence network analysis revealed complex interactions among ARGs, MGEs, and microbial communities during composting. The network suggested that *Firmicutes*, *Proteobacteria*, *Actinobacteria*, and *Bacteroidetes* could represent the primary potential host, which aligns with previous studies (Zhang et al., 2021; Qiu et al., 2022). A key finding was the heterogeneity in host range: *intI1* and *tetG* were linked to a wide range of genera, pointing to a broad dissemination potential (Zhang et al., 2023); whereas the narrow associations of *qnrC* and *Tn916/1545*, may be attributed to competitive inhibition by compost inoculants (Wang et al., 2021). In this study, genera such as *Lysinibacillus*, *Luteimonas*, *Brachybacterium*, and *Brevibacterium* were significantly associated with multiple target genes. This finding supports the view that the widespread occurrence of multi-gene potential hosts can explain the simultaneous presence of multiple ARGs in the environment (Li et al., 2022). By the end of composting, the abundances of genera in *Firmicutes* and *Actinobacteria* had decreased, especially in the ST group, which correlates with the observed decline in most ARGs and MGEs, underscoring the link between host dynamics and gene fate (Qiu et al., 2022). Notably, *ermX* and *sul2* shared a high degree of associated genera, suggesting potential co-transmission (Li et al., 2022). Their enrichment in the later stages of composting likely stems from the increased abundance of these shared host genera (Liu et al., 2023).

MGEs exhibited distinct association patterns. While *intI1* was correlated with more genera than *intI2*, their overlapping genera (e.g., *Luteimonas*) and co-occurrence with transposons like *Tn916/1545* suggests a possible synergistic role in ARG dissemination (Lima et al., 2020). Furthermore, pathogenic bacteria acting as key hosts pose potential risks (Wei et al., 2022). Several pathogenic bacteria were identified as multi-ARG-MGE potential hosts: *Corynebacterium* and *Pseudomonas* were associated with multiple ARGs and MGEs, such co-occurrence could potentially facilitate gene transfer (Zhou et al., 2021). While *Corynebacterium* was effectively removed by the end of composting, *Pseudomonas* and *Bacillus* (pathogenic genera, Wei et al., 2022) proliferated to varying degrees. This proliferation was significantly suppressed by the ST treatment, highlighting an advantage of the Sta2 inoculant.

## 5 Conclusion

Co-inoculation with Sta2 and commercial bacterial agents during composting effectively extended the high-temperature phase above 55 °C by 7 days, significantly reduced the relative abundances of most target genes, and enhanced the removal of specific ARGs (*tetG, sul1, ermQ,* and *aac(6′)-Ib-cr*) and MGEs (*intI1* and *intI2*) by 4.8%–48.4%, while also inhibiting the enrichment of *sul2* and *ermX*. Bacterial community succession and MGE-mediated horizontal gene transfer served as the core drivers for ARG reduction, with variations in *intI1* and *Firmicutes* abundance exerting the most significant impact. Network analysis identified several genera, such as *Lysinibacillus*, *Luteimonas*, *Brachybacterium*, and the pathogen *Corynebacterium,* as potential hosts for multidrug-resistant genes. Furthermore, the strategy suppressed pathogens such as *Corynebacterium* and *Pseudomonas*, thereby enhancing compost biosafety. Overall, this approach enhanced ARG removal through sustained high-temperature sterilization, microbial community restructuring, and blocking ARG–MGE transmission, providing an effective solution for controlling ARG pollution and improving biosafety in livestock manure composting.

## Data Availability

The sequence data from this study have been submitted to the NCBI SRA under the accession number PRJNA1301025 (https://www.ncbi.nlm.nih.gov/bioproject/PRJNA1301025).
