# Supplementary material for: Co-inoculation with Streptomyces thermovulgaris and commercial microbial agents enhances the reduction of antibiotic resistance genes in cattle manure composting: driving mechanisms involving microbial communities and mobile genetic elements
Source: Front Microbiol. 2025 Nov 27;16:1688304. doi: 10.3389/fmicb.2025.1688304 (PMC12695823; doi:10.3389/fmicb.2025.1688304)
Supplement: Supplementary file 1 [file Table_1.DOCX]

Co-inoculation with *Streptomyces thermovulgaris* and Commercial Microbial Agents Enhances the Reduction of Antibiotic Resistance Genes in Cattle Manure Composting: Driving Mechanisms of Microbial Communities and Mobile Genetic Elements

**Supplementary Material**

**1 Basic characteristics of composting materials**

Table S1 Basic characteristics of composting materials (dry weight)

| Raw material for testing | Organic carbon (%) | Nitrogen (%) | Carbon to nitrogen ratio (C/N) | Moisture content (%) |
| --- | --- | --- | --- | --- |
| Sawdust | 45.7 | 0.35 | 130.6 | 19.21 |
| Broiler breeding bedding | 28.5 | 2.79 | 10.2 | 16.27 |
| Cattle manure | 29.7 | 2.60 | 11.4 | 88.26 |

**2 DNA concentration of two treatments**

Table S2 DNA Concentration of two treatments at different time points

| Treatment | 0 | 3 | 10 | 20 | 35 |
| --- | --- | --- | --- | --- | --- |
| CK | 26.86±1.29 | 30.47±4.31 | 54.85±12.47 | 68.04±10.63 | 81.61±0.83 |
| ST | 26.86±1.29 | 46.03±0.02 | 75.69±0.74 | 69.84±15.99 | 104.93±0.05 |

Note: DNA concentration (ng/μL) is expressed as mean ± SD (n=2). Numbers 0-35 denote compost sampling time points.

**3 PCR and qPCR methods**

**3.1 PCR and qPCR primer sequences, fragment sizes, and annealing temperature**

Table S3 PCR and qPCR primer sequences, fragment sizes, and annealing temperatures

| Gene | Primer sequence (5'-3') | Size(bp) | Annealing Temp. (℃) |  |
| --- | --- | --- | --- | --- |
| *tetB/P* | F: AAAACTTATTATATTATAGTC  R: TGGAGTATCAATAATATTCAC | 169 | 46 |  |
| *tet C* | F: GCGGGATATCGTCCATTCCG  R: GCGTAGAGGATCCACAGGACG | 207 | 59 |  |
| *tetG* | F: GCAGAGCAGGTCGCTGG  R: CCYGCAAGAGAAGCCAGAAG | 134 | 54 |  |
| *tetQ* | F: AGAATCTGCTGTTTGCCAGTG  R: CGGAGTGTCAATGATATTGCA | 169 | 55 |  |
| *tetT* | F: AAGGTTTATTATATAAAAGTG  R: AGGTGTATCTATGATATTTAC | 169 | 46 |  |
| *tetW* | F: GAGAGCCTGCTATATGCCAGC  R: GGGCGTATCCACAATGTTAAC | 168 | 56 |  |
| *tetX* | F: CAATAATTGGTGGTGGACCC  R: TTCTTACCTTGGACATCCCG | 468 | 55 |  |
| *sul1* | F: CGGCGTGGGCTACCTGAACG  R: GCCGATCGCGTGAAGTTCCG | 433 | 60 |  |
| *sul2* | F: GCGCTCAAGGCAGATGGCATT  R: GCGTTTGATACCGGCACCCGT | 293 | 59 |  |
| *dfrA7* | F: AAATGGCGTAATCGGTAATG  R: GTGAACAGTAGACAAATGAAT | 304 | 51 |  |
| *ermQ* | F: CACCAACTGATATGTGGCTAG  R: CTAGGCATGGGATGGAAGTC | 154 | 55 |  |
| *ermX* | F: GAGATCGGRCCAGGAAGC  R: GTGTGCACCATCGCCTGA | 488 | 58 |  |
| *gyrA* | F: CGATGTCGGTCATTGTTGGC  R: ATACCTACGGCGATACCGGA | 455 | 61 |  |
| *parC* | F: GCCTAAACAACGCACGGAAA  R: TGACACGGGAGGTAACCAGA | 432 | 53 |  |
| *qnrA* | F: AGGATTGCAGTTTCATTGAAAGC  R: TGAACTCTATGCCAAAGCAGTTG | 138 | 53 |  |
| *qnrC* | F: TTCGATCGGACTGCTTGTGG  R: AACACATGGTGCAGGGGATT | 438 | 53 |  |
| *qnrS* | F: CCCCATGCCCGAAGTTATCA  R: ACTGCTTGGAGTGTGTTGGT | 457 | 53 |  |
| *aac(6')-Ib-cr* | F: TTGCGATGCTCTATGAGTGGCTA  R: CTCGAATGCCTGGCGTGTTT | 482 | 55 |  |
| *intI1* | F: AGCATTACCCAACCGAAAGT  R: TGTCAGCAAGATAGCCAGAT | 473 | 60 |  |
| *intI2* | F: GTTATTTTATTGCTGGGATTAGGC  R: TTTTACGCTGCTGTATGGTGC | 166 | 55 |  |
| *Tn916/1545* | F: TCCTACAGCGACAGCCAGTGA  R: TGCGTTGCTTTGGTCTGCTGGT | 174 | 55 |  |
| *ISCRI* | F: CGCCCACTCAAACAAACG  R: GAGGCTTTGGTGTAACCG | 452 | 54 |  |
| 16SrRNA | F: CCTACGGGAGGCAGCAG  R: ATTACCGCGGCTGCTGG | 193 | 55 |  |

**3.2 Target gene screening and quantification**

Table S4 Overview of target gene screening and quantitative analysis

| Category | Gene Name | PCR Screening | PCR Detection Result | qPCR Quantification |
| --- | --- | --- | --- | --- |
| tetracycline | *tetB/P* | √ | Negative | × |
|  | *tetC* | √ | Negative | × |
|  | *tetG* | √ | Positive | √ |
|  | *tetQ* | √ | Positive | √ |
|  | *tetT* | √ | Negative | × |
|  | *tetW* | √ | Positive | √ |
|  | *tetX* | √ | Negative | × |
| sulfonamide | *sul1* | √ | Positive | √ |
|  | *sul2* | √ | Positive | √ |
|  | *dfrA7* | √ | Negative | × |
| macrolide | *ermQ* | √ | Positive | √ |
|  | *ermX* | √ | Positive | √ |
| quinolone | *gyrA* | √ | Negative | × |
|  | *parC* | √ | Negative | × |
|  | *qnrA* | √ | Negative | × |
|  | *qnrC* | √ | Positive | √ |
|  | *qnrS* | √ | Negative | × |
| aminoglycoside | *aac(6’)- Ib-cr* | √ | Positive | √ |
| MGEs | *intI1* | √ | Positive | √ |
|  | *intI2* | √ | Positive | √ |
|  | *Tn916/1545* | √ | Positive | √ |
|  | *ISCRI* | √ | Negative | × |

**3.3 SI: PCR/qPCR reaction system and conditions**

The reaction system had a total volume of 20 μL and consisted of 1 μL genomic DNA (10 ng/μL), 10 μL of the respective master mix (PCR: 2×Taq PCR Mix, Tiangen; qPCR: BlasTaq^TM^ 2×qPCR MasterMix, ABM), 0.25 μL forward and reverse primers (TsingKe), and 8.5 μL ddH_2_O.

The PCR reaction conditions were as follows: pre-denaturation at 94°C for 5 min, 30 cycles of denaturation at 94°C for 45 s, annealing as in Table S3 for 30 s, extension at 72°C for 60 s, and a final extension at 72°C for 7 min.

The qPCR reaction underwent pre-denaturation at 95°C for 3 min, 40 cycles of denaturation at 95°C for 15 s, annealing as in Table S3 for 60 s, extension at 72°C for 32 s, and a final step at 65°C for 5 s.

**4 Changes in bacterial communities during composting**

**4.1 Principal coordinate analysis of bacterial community changes**

Fig. S1: Principal coordinate analysis of bacterial community changes.

**
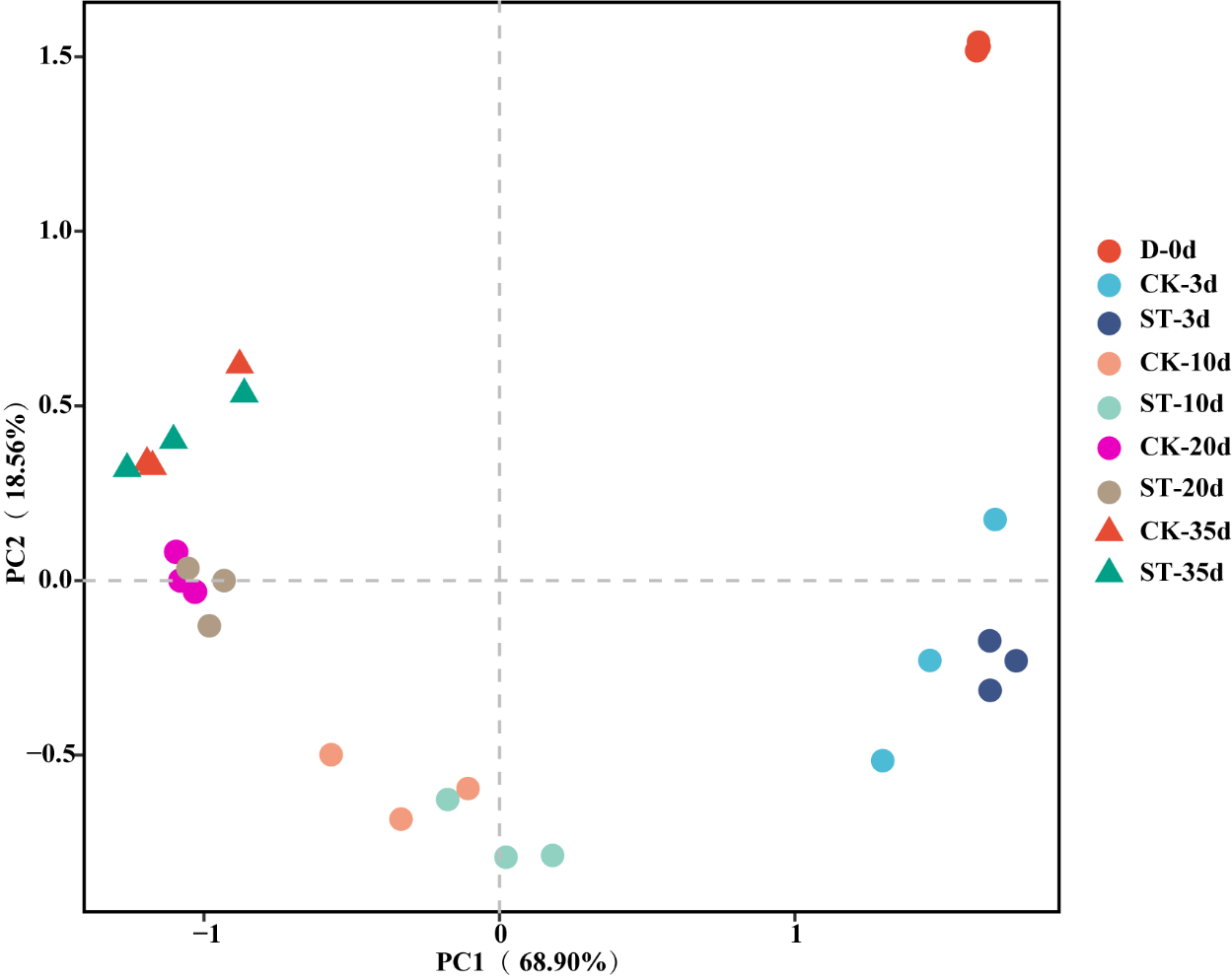
**

Note: D-0 represents the initial sample at composting start, CK is the control group, and ST is the treatment group. 0, 3, 10, 20, and 35 are time points in the composting process. The same applies below.

**4.2 Changes in abundance of major bacterial phyla**

Table S5 Relative abundance of major bacterial phyla (%)

| Phylum | Group | 0 | 3 | 10 | 20 | 35 |
| --- | --- | --- | --- | --- | --- | --- |
| *Firmicutes* | CK | 42.6±0.5 | 61.2±8.9 | 21.9±4.0 | 6.4±1.7 | 4.1±1.3 |
|  | ST |  | 58.4±3.5 | 29.5±4.4 | 7.6±1.3 | 3.7±0.4 |
| *Proteobacteria* | CK | 12.9±1.7 | 8.6±4.2 | 24.6±2.8 | 25.3±3.5 | 27.9±4.5 |
|  | ST |  | 6.3±0.2 | 17.8±3.8 | 27.4±2.9 | 27.8±0.4 |
| *Actinobacteria* | CK | 17.2±3.9 | 10.5±2.5 | 14.9±0.6 | 17.4±2.5 | 14.7±3.5 |
|  | ST |  | 13.4±1.3 | 13.0±2.1 | 23.3±0.8 | 15.7±3.2 |
| *Bacteroidetes* | CK | 15.0±4.1 | 1.9±1.0 | 10.5±0.9 | 15.3±1.6 | 18.5±0.3 |
|  | ST |  | 3.4±0.6 | 8.4±2.3 | 15.5±1.2 | 20.2±1.3 |
| *Planctomycetes* | CK | 1.1±0.2 | 0.4±0.3 | 6.8±2.3 | 12.7±3.6 | 12.4±3.1 |
|  | ST |  | 0.4±0.0 | 5.3±1.3 | 6.0±1.5 | 12.7±1.0 |
| *Chloroflexi* | CK | 0.6±0.05 | 0.5±0.2 | 3.3±0.7 | 5.5±2.2 | 6.7±1.5 |
|  | ST |  | 0.4±0.1 | 5.4±1.9 | 6.2±2.1 | 4.1±0.4 |

Note: Values represent mean ± SD (n = 3) for both treatments at various time points.

**4.3 Relative abundances of three types of bacterial communities at different time points**

Table S6 Total relative abundance of genus clusters

| Cluster | Group | 0 | 3 | 10 | 20 | 35 |
| --- | --- | --- | --- | --- | --- | --- |
| A | CK | 1.8±0.3 | 78.5±4.4 | 28.9±2.5 | 10.4±4.5 | 6.9±3.0 |
|  | ST |  | 73.1±0.9 | 39.1±4.6 | 11.4±1.9 | 5.4±0.6 |
| B | CK | 79.7±3.1 | 14.4±2.2 | 6.1±1.1 | 2.4±0.5 | 1.5±0.2 |
|  | ST |  | 19.4±0.9 | 6.8±1.7 | 2.9±1.1 | 1.1±0.1 |
| C | CK | 18.5±2.8 | 7.1±2.9 | 65.0±3.8 | 87.2±4.9 | 91.6±3.5 |
|  | ST |  | 7.5±1.7 | 54.1±3.2 | 85.7±2.5 | 93.5±0.5 |

Note: The top 35 genera by relative abundance were clustered into three groups (A/B/C). Data are shown as mean ± SD (n=3) for the total relative abundance of genus clusters A (8 genera), B (8 genera), and C (19 genera).

**4.4 Relative abundances of key functional genera**

Table S7 Relative abundances of key functional genera

| Genera | Group | 0 | 10 | 20 | 35 |
| --- | --- | --- | --- | --- | --- |
| *Luteimonas* | CK | 4.7±0.2 |  |  | 3.0±1.9 |
|  | ST |  |  |  | 3.8±1.6 |
| *Pusillimonas* | CK | 2.6±1.3 |  |  | 2.6±0.4 |
|  | ST |  |  |  | 2.3±1.2 |
| *Pseudomonas* | CK | 2.7±0.2 |  |  | 6.0±2.0 |
|  | ST |  |  |  | 2.3±0.7 |
| *Methanosarcina* | CK | 3.6±0.4 | 7.7±1.1 |  | 2.1±1.1 |
|  | ST |  | 11.4±3.5 |  | 9.8±7.6 |
| *Actinomadura/*  *Longispora* | CK | 0.15±0.12 |  | 12.6±4.7 | 5.4±1.8 |
|  | ST |  |  | 19.5±4.0 | 5.8±1.2 |
| *Steroidobacter* | CK | 0.2±0.1 |  |  | 11.4±5.3 |
|  | ST |  |  |  | 10.8±5.3 |
| *Castellaniella* | CK | 0.04±0.0 |  |  | 7.8±3.8 |
|  | ST |  |  |  | 7.4±2.3 |
| *Chryseolinea* | CK | 0.19±0.05 |  |  | 25.1±3.7 |
|  | ST |  |  |  | 23.6±7.2 |

Note: Values are mean ± SD (n = 3) of the relative abundance for the key functional genera.

**5 Correlation of major bacterial phyla, physicochemical factors, and target genes**

Fig. S2. Heatmap of Spearman rank correlation analysis among major bacterial phyla, physicochemical factors, and target genes (**P*<0.05, ***P*<0.01).

**
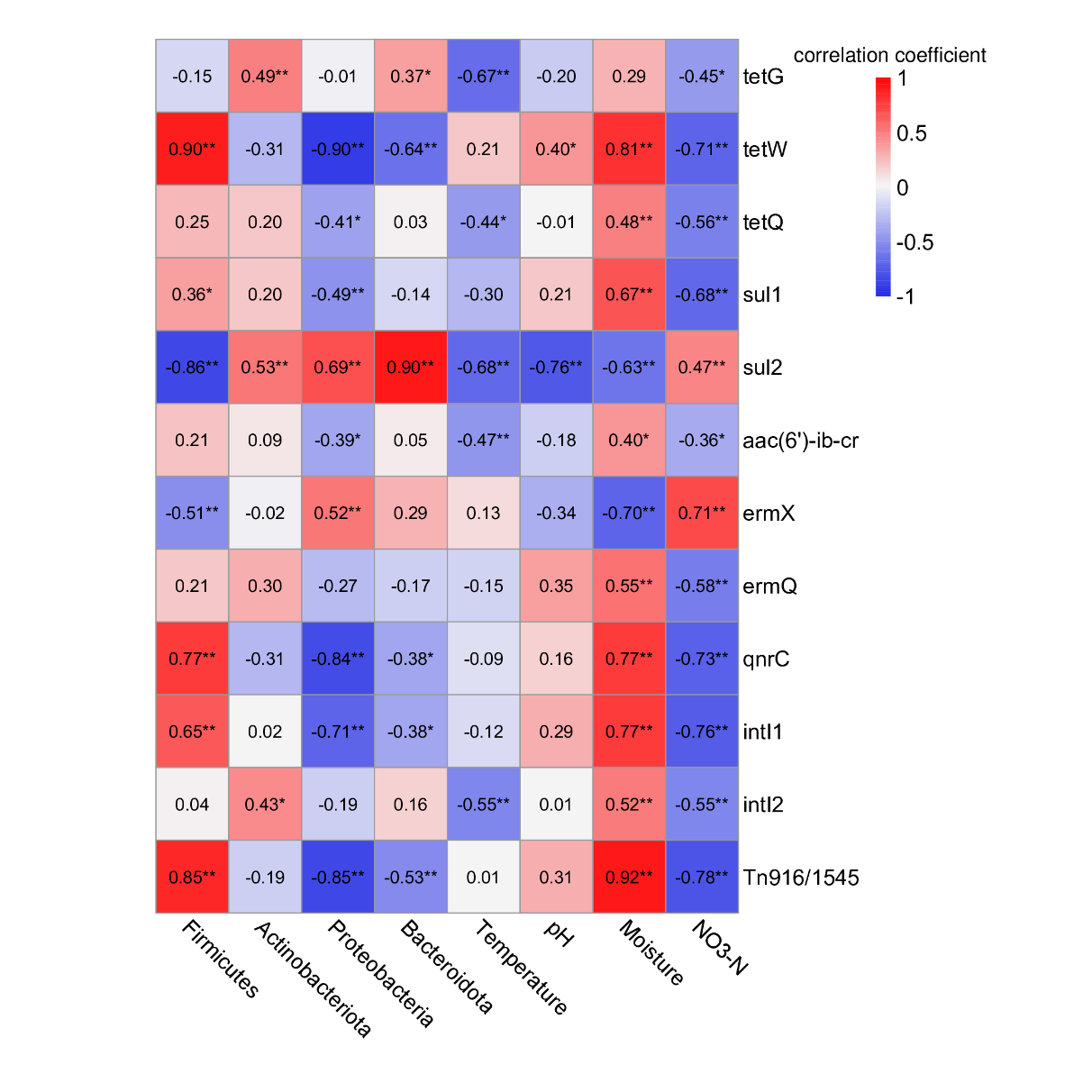
**

**6** **Correlation analysis of ARGs and MGEs**

Fig. S3 Pearson correlation analysis of correlations between ARGs and MGEs (**P*<0.05, ***P*<0.01).

**
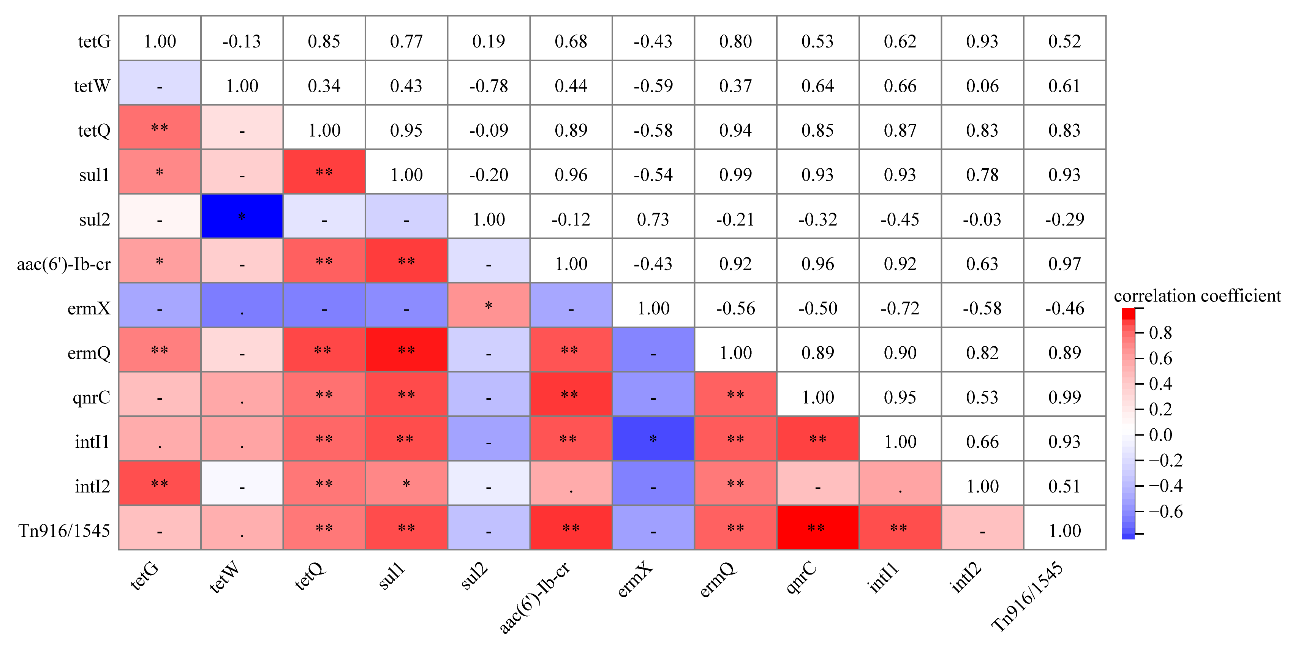
**
